# Highly Sensitive and Linear Resonator-Based Biosensor for White Blood Cell Counting: Feasible Measurement Method and Intrinsic Mechanism Exploration

**DOI:** 10.3390/bios14040180

**Published:** 2024-04-07

**Authors:** Yi-Ke Wang, Bo-Wen Shi, Jun-Ming Zhao, Yan-Xiong Wang, Yan-Feng Jiang, Gang-Long Yang, Xiao-Dong Gao, Tian Qiang

**Affiliations:** 1 School of Internet of Things Engineering, Institute of Advanced Technology, Jiangnan University, Wuxi 214122, China; 1038210220@stu.jiangnan.edu.cn (Y.-K.W.); 1038210214@stu.jiangnan.edu.cn (B.-W.S.); 1038210218@stu.jiangnan.edu.cn (J.-M.Z.); 7231923001@stu.jiangnan.edu.cn (Y.-X.W.); jiangyf@jiangnan.edu.cn (Y.-F.J.); 2 State Key Laboratory of Biochemical Engineering, Institute of Process Engineering, Chinese Academy of Sciences, Beijing 100190, China; glyang@ipe.ac.cn; 3 Key Laboratory of Biopharmaceutical Preparation and Delivery, Chinese Academy of Sciences, Beijing 100190, China; 4 School of Biotechnology, The Key Laboratory of Carbohydrate Chemistry and Biotechnology, Ministry of Education, Jiangnan University, Wuxi 214122, China

**Keywords:** highly sensitive, permittivity-inspired, linear response, microwave biosensor, WBC counting

## Abstract

Since different quantities of white blood cells (WBCs) in solution possess an adaptive osmotic pressure of cells, the WBCs themselves and in solution have similar concentrations, resulting in them having similar dielectric properties. Therefore, a microwave sensor could have difficulty in sensing the quantity variation when WBCs are in solution. This paper presents a highly sensitive, linear permittivity-inspired microwave biosensor for WBCs, counting through the evaporation method. Such a measurement method is proposed to record measurements after the cell solution is dripped onto the chip and is completely evaporated naturally. The proposed biosensor consists of an air-bridged asymmetric differential inductor and a centrally located circular fork-finger capacitor fabricated on a GaAs substrate using integrated passive fabrication technology. It is optimized to feature a larger sensitive area and improved Q-factor, which increases the effective area of interaction between cells and the electromagnetic field and facilitates the detection of their changes in number. The sensing relies on the dielectric properties of the cells and the change in the dielectric constant for different concentrations, and the change in resonance properties, which mainly represents the frequency shift, corresponds to the macroscopic change in the concentration of the cells. The microwave biosensors are used to measure biological samples with concentrations ranging from 0.25 × 10^6^ to 8 × 10^6^ cells per mL in a temperature (26.00 ± 0.40 °C) and humidity (54.40 ± 3.90 RH%) environment. The measurement results show a high sensitivity of 25.06 Hz/cells·mL^−1^ with a highly linear response of *r*^2^ = 0.99748. In addition, a mathematical modeling of individual cells in suspension is performed to estimate the dielectric constant of individual cells and further explain the working mechanism of the proposed microwave biosensor.

## 1. Introduction

A complete blood count is a standard laboratory and typical clinical test that is important in assessing overall human health and providing information about the disease. A complete blood count can provide information about blood cell production, serve as an indicator of health, and help diagnose several disorders by assessing a specific blood cell count. Red blood cells, white blood cells, and platelets are the three main types of blood cells [1]. A white blood cell (WBC) count is an essential part of a complete blood count. WBCs, derived from pluripotent hematopoietic stem cells, are an important part of the host’s defense system against bacteria, viruses, and other invasions [2]. WBCs can pass through the capillary walls through deformation and concentrate at the site of invasion to surround and engulf the germs when germs invade the body. When the number of WBCs in our body increases and exceeds the average level, it is usually caused by bacterial infections, tissue damage, and inflammatory diseases (rheumatoid arthritis, lupus erythematosus, and other immune system sensitivities). Conversely, it is usually associated with bone marrow deficiency, certain viral infections, and severe bacterial infections [3]. In addition, a highly significant negative correlation at lower plasma levels is demonstrated between WBC counts and WBC ascorbic acid content, which in turn may be used to assess the body’s nutritional status [4]. 

A WBC count can predict several other diseases besides indicating whether the body is healthy or not as follows. For example, after researching the WBC count of a large number of coronary artery disease patients and comparing them to a corresponding control group, William B. Kannel et al. found that the WBC counts of cardiovascular disease patients are significantly elevated. The degree to which white blood cells are elevated within the normal range is a marker of the increased risk of cardiovascular disease [5]. The total WBC count has been proven to be an independent predictor of death/myocardial infarction and is of great significance for the risk assessment of coronary heart disease. It may facilitate a more in-depth examination and treatment of patients with cardiovascular disease if the WBC count is abnormal [6]. Additionally, blood tests and physical examinations were conducted at a general hospital health checkup center to analyze the relationship between WBC counts. The results show that the prevalence of metabolic syndrome increased with an increasing WBC count in people [7]. A WBC count can also predict diabetes independently of two previously identified predictors of diabetes: insulin resistance and insulin secretion [8]. Nowadays, with an aging population, the continuing pressure of obesity and overweight, cardiovascular disease morbidity, and mortality are at the top of the list, and often patients are subject to morbidity and have life-threatening conditions before they are even detected. Therefore, the predictive value of a WBC count in cardiovascular disease has indicated the need for routine measurements of WBCs. In this way, we will be able to detect the risk of disease to a certain extent and thus have time to take preventive measures. 

Until the early 1970s, the only universally accepted method of WBC counting was the microscopic examination of Romanovsky-stained blood smears by trained professionals [9]. However, over time, many problems have emerged with this traditional method, such as high thresholds, inaccurate measurements, low sensitivity, and poor specificity. As the requirement for the accuracy of cell counting results continues to increase, automated methods have emerged. Automation has the advantages of high-throughput, reducing imprecision (essentially due to counting a larger number of cells), and further generating clinically useful parameters such as immature reticulocyte fractions and reticulocyte indices [10]. Currently, available methods are automated differential counters and hematology analyzers, while WBC counting using hematology analyzers is widely recognized. Automated hematology analyzers are usually classified as either laser scatter or electrical impedance based on the mechanism. Wallace H. Coulter and Joseph R. Coulter discovered that when blood cells pass through an inductive zone, their pulse signal changes due to a change in current direction or resistance. These signals can be measured and converted into data that help identify the different types of cells and their numbers in the blood. Based on this principle, a new fully automated cell counter was designed, bringing a refreshing change to medical research and diagnosis [11]. However, cell analyzers using spectral scattering require that the cells be stained first, and different types of white blood cells are stained to scatter the scattered light of different sizes and directions [12]. However, the dyeing steps are too complicated and require specialized skills. Research is raising the possibility of new parameters to be applied to automated cell analyzers, such as the reticulocyte index, which can extend differential counting beyond the five normal WBC populations [13]. However, operators need to have specialized theoretical knowledge to analyze the results. And, using the artificial intelligence (AI) model YOLOv5 to detect blood smear images helps to attain WBC counts [14]. There are also studies proposing methods for the automated counting of microscope images, employing convolutional neural network regressors and a successful convolutional architecture with residual connectivity to achieve accurate and efficient cell counting [15]. The image counting method requires a corresponding application program, which requires a large amount of data support and complex machine algorithm training. Additionally, automated blood analyzers have many limitations. They require regular calibration and maintenance by specialized personnel with expensive costs and bulky equipment, which may be costly to purchase and maintain for some small medical institutions or laboratories. Moreover, this test cannot be used in certain special environments. A microfluidic single-cell impedance cytometer can perform differential WBC counting. When the cell passes through the electric field in the microfluidic chip, the different parameters such as the size, shape, and dielectric properties of the cell will lead to changes in the electrical impedance signal, based on which the cell and other particles can be distinguished and the cells can be counted, and parameters such as the number of cells and their concentration can be calculated by algorithms and related software [16]. For example, using the spatiotemporal properties of impedance-sensing signals, an impedance-based camera-less multimodal electromechanical flow cytometry framework for dynamic high-dimensional intrinsic measurements, has been proposed, enabling integrated, real-time, accurate, label-free, high-dimensional, intrinsic biophysical characterization of single cells [17]. Glutaraldehyde fixation at varying levels is able to generate a class of fixed red blood cells. After being mapped against model red blood cell types, it facilitates the microfluidic device to recognize and determine the propagation of subcellular biophysical information and its dielectrophoretic cross-frequency [18]. A novel microdevice with a coplanar differential electrode structure has been proposed to be able to accurately resolve the spatial location of single cells without limiting techniques such as additional sheath fluid or narrow microchannels, capable of distinguishing between cells and particles [19]. An AgPDMS-based microfluidic impedance cytometry device was able to be used for a single-cell analysis of tumor cells through the characterization of some electrical parameters [20]. A bipolar electrode (BPE)-based sheath-less focusing, switching, and tilt angle standing surface acoustic wave for sorting cells and particles in a continuous flow has been proposed to provide a powerful and unique method for cell-sorting applications [21]. Certain biosensors have good specific recognition and can directly detect biological samples, such as biosensors that directly detect tumor markers [22]. Biosensors have also been widely used in cell counting, such as a blood cell counter sensor implemented using MEMS technology [23] and a WBC count on a smartphone paper electrochemical sensor [24]. Microwave sensors are widely used for their high sensitivity, non-contact detection, real-time measurement, and fast response, which can convert the change in the dielectric properties of the measured substance into an electrical signal and have been proven to have great potential in bio-sensing [25,26,27]. They can be used for real-time blood glucose detection [28,29], as well as the real-time detection of bacterial concentrations [30,31]. Therefore, microwave biosensors could be a promising candidate for the application of WBC counting. We are committed to developing a small, simple, fast platform for WBC counting with microwave biosensors.

**Figure 1:**
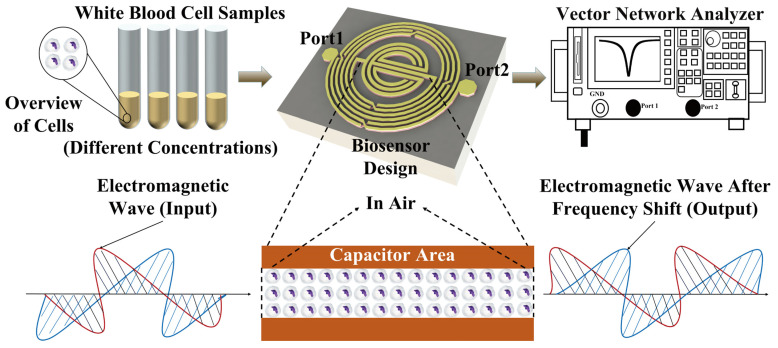
Conceptual representation of the experiments to detect different concentrations of white blood cells.

This study presents a WBC counting method based on microwave biosensors constructed for the first time with the main objective of using a microwave biosensor for a single sample to demonstrate the feasibility of cell counting, constituting some preliminary work, as shown in Figure 1. The future application expects to be used in measurement or calibration devices in hospitals or research facilities. Several sets of WBC solutions with different concentrations are measured in the experiment. Each set of concentration solution is added (1 μL) to the integrated passive device (IPD) chip using a pipette. When the solution on the chip is completely naturally evaporated, the number of cells left on the chip will vary with different concentrations of the solution. Cells entering the sensor-sensitive area will alter the dielectric constant in the space and the distribution of its electromagnetic field, thereby comprehensively changing the overall transmission characteristics of the sensor and causing variations in the scattering parameters. These changes corresponding to the cells are taken as the experimental results, and a linear sensing response is finally obtained. 

## 2. Materials and Methods

### 2.1. Design and Optimization of the Microwave Sensor

**Figure 2:**
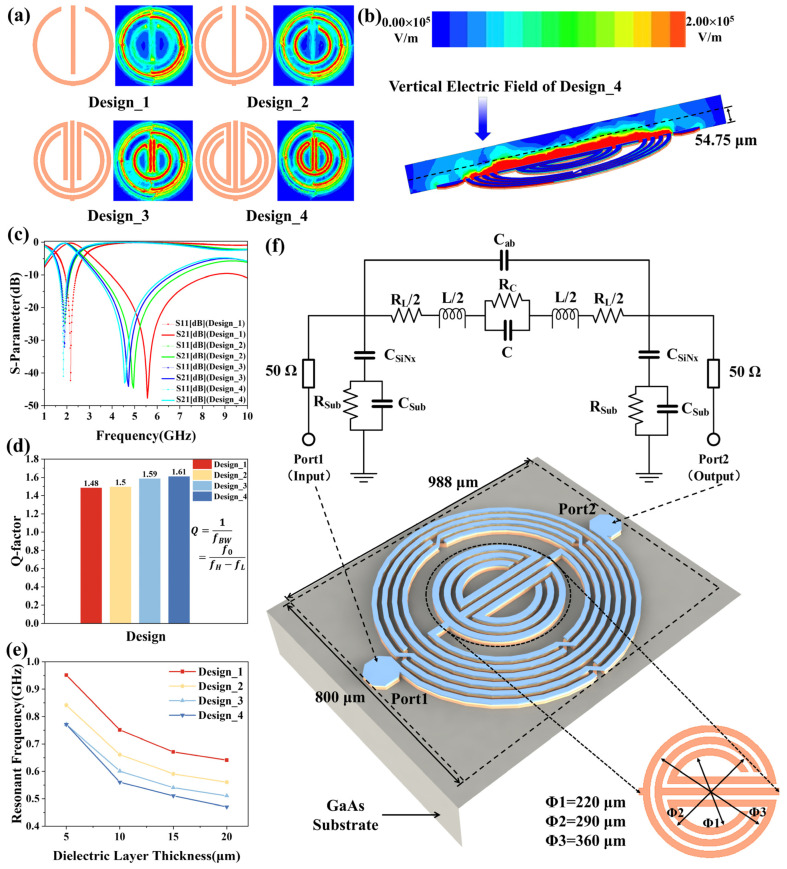
Optimization of the microwave sensor and the proposed microwave sensor structure. (**a**) The intermediate capacitor structure and the electric field of the designs; (**b**)vertical electric field of Design_4; (**c**) simulated S−parameters; (**d**) Q−factor of the designs; (**e**) relationship between resonant frequency and thickness of dielectric layer; (**f**) 3D structure and equivalent circuit of the proposed sensor.

Based on the Agilent Advanced Design System (ADS), we design the layout of the microwave biosensor and optimize the value of the inductor and the capacitor to operate at a low center frequency that increases the electric field penetration depth in the WBC sample, improving the sensitivity of the sensor. In addition, the design performance of biosensors is improved by increasing the sensitive area of sensors, making it easier to detect changes in the number of white blood cells. Moreover, when the electromagnetic field propagates in the biological medium, the strength of the electric field will slowly decay. When a large number of cells are detected, they will be stacked together and have a certain thickness, so the penetration depth of the sensor is a concern. The penetration depth can be determined by [32,33]

(1) $$D_{p}=\frac{\lambda_{0}}{2\pi {\left(2\varepsilon^{\prime }\right)}^{\frac{1}{2}}}{\left\{\left[1+\left(\frac{\varepsilon^{\prime }}{\varepsilon^{\prime \prime }}\right)\right]-1\right\}}^{-\frac{1}{2}},$$

where $\lambda_{0}\left(=c/f\right)$ is the wavelength of the microwave in free space, c is the speed of the electromagnetic wave in free space, and f is the frequency. Additionally, the concentrated electric field strength and penetration depth are critical factors to achieve high sensitivity while keeping the sample volume low [34]. Therefore, the capacitor in the middle is optimized four times, resulting in four designs to generate high capacitance and larger electric fields. As shown in Figure 2a, the sensitive area of Design_4 is the largest. Figure 2c,d represent the simulated S-parameters and Q-factor of the four designs. The resonant frequency of Design_4 is the lowest, making its penetration depth the greatest, which means that it has high sensitivity. What is more, a higher Q-factor is better for detecting minimal changes in permittivity [35]. From Figure 2d, it can be seen that the Q-factor of Design_4 is the highest. Figure 2e shows the resonant frequency variation in the four resonator designs for different dielectric thicknesses. After investigation, the dielectric constant of the simulated dielectric layer as well as the loss tangent values are taken from other cells due to the unknown dielectric constant of WBC [36]. The roughly calculated sensitivities of Design_1, Design_2, Design_3, and Design_4 are 0.021 GHz/μm, 0.019 GHz/μm, 0.017 GHz/μm, and 0.020 GHz/μm, respectively. Accordingly, a trade-off should be made between the performance of the size of the sensitive area, Q-factor, sensitivity, and the performance of the self-resonant frequency. Finally, Design_4 is chosen for the experiment as it has the largest sensitive area, a good Q-factor, and a low resonant frequency compared with the other designs.

### 2.2. Analysis of the Proposed Microwave Sensor Structure

The proposed microwave sensor is fabricated using IPD technology on a GaAs substrate, mainly including an L-C resonator, as depicted in Figure 2f. It comprises an asymmetrical spiral inductor, a circinate capacitor, and five air bridges. The GaAs substrate has a dielectric constant ε of 12.85, a loss tangent tan δ of 0.00028, and height of 200 µm. Additionally, the inner-digital non-crossing circinate capacitor can achieve a concentrated electric field, contributing to the total capacitance. The resonant frequency of the microwave resonator is also determined by the total capacitance and the total inductance, which is calculated as follows:

(2) $$f=\frac{1}{2\pi \sqrt{L_{\mathrm{total}}C_{\mathrm{total}}}}.$$

Figure 2f also shows the equivalent circuit of the proposed resonator. The capacitance introduced by the SiNx layer is denoted as C_SiNx_. The C_ab_ is the fringing capacitance between inductor turns. C_Sub_ and R_Sub_ represent shunt capacitance and resistance between the trace and the substrate, respectively. The inductance of the external circular inductor can be determined by [37]

(3) $$L_{\mathrm{total}}=\frac{\mu_{0}n^{2}d_{\mathrm{avg}}c_{1}}{2}\left(\ln\left(\frac{c_{2}}{\rho }\right)+c_{3}\rho +c_{4}\rho^{2}\right),$$

where the coefficients $c_{1}$, $c_{2}$, $c_{3}$, and $c_{4}$ relate to the layout, and for the circular inductor, their values are 1.00, 2.16, 0.00, and 0.20, respectively. Furthermore, n and $d_{\mathrm{avg}}$ represent the number of turns and average diameter of the circular inductor. And, ρ means the fill ratios that can be expressed by

(4) $$\rho =\frac{d_{\mathrm{out}}-d_{\mathrm{in}}}{d_{\mathrm{out}}+d_{\mathrm{in}}},$$

where $d_{\mathrm{out}}$, and $d_{\mathrm{in}}$ represent the outer diameter and the inner diameter, respectively. Furthermore, determined by the circular finger length $L_{C}$, free space permittivity ($\varepsilon_{0}$ = 8.854 × 10^−12^ F/m), and permittivity of the GaAs substrate ($\varepsilon_{\mathrm{sub}}$ = 12.85 F/m), the inner circinate capacitor can be calculated using the equation below [38]

(5) $$C_{\mathrm{total}}=\left[\varepsilon_{0}\varepsilon_{\mathrm{sub}}\left(\frac{1+\varepsilon_{s}}{2}\right)\frac{K\left(\sqrt{1-k^{2}}\right)}{K\left(k\right)}+\varepsilon_{0}\frac{t}{a}+{\left\{\frac{K(k)}{\varepsilon_{0}K\left(\sqrt{1-k^{2}}\right)}\right\}}^{-1}\right]L_{C},$$

where k(=a/b) and K(k) are the elliptic integrals of the first kind, and the concentrated electric field intensity of the proposed biosensor is simulated through a high-frequency structure simulator (HFSS) at the operating resonant frequency of 1.28 GHz. The red color in the middle area is the most sensitive region of the resonator, indicating the maximum electric field concentrated at the center for the microwave biosensor application.

### 2.3. Sample Preparation and Characterization

**Figure 3:**
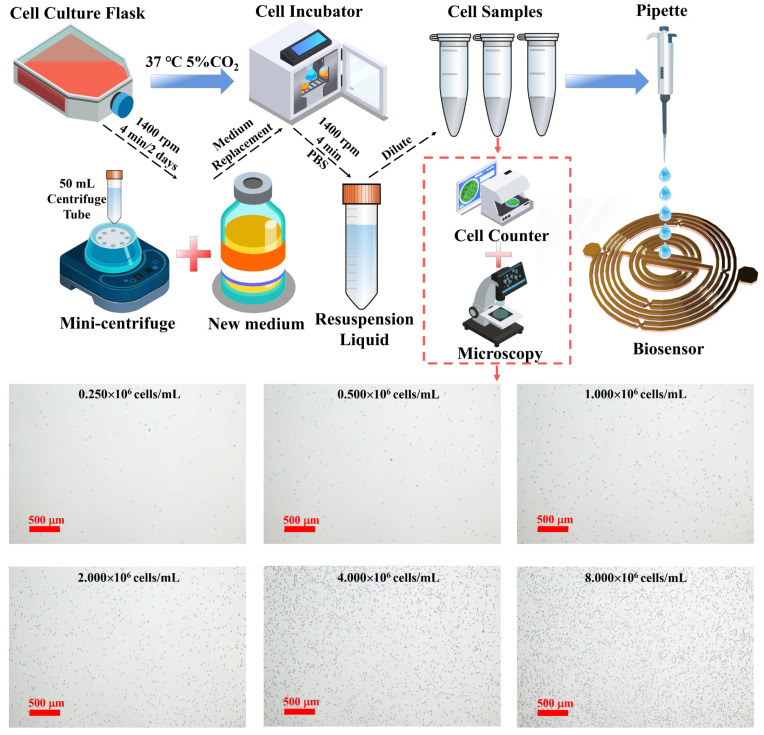
Sample preparation process and micrographs of cells at different concentrations.

Figure 3 represents the process of cell sample preparation. The human WBC line K562 is obtained from the Cell Bank of the Chinese Academy of Sciences, Shanghai. Cells are cultured in RPMI 1640 medium (Biological Industries, Beit Haemek, Israel) supplemented with 10% fetal bovine serum (Biological Industries, Beit Haemek, Israel) and 1% penicillin–streptomycin (Biological Industries, Beit Haemek, Israel). Cells are maintained in a humidified atmosphere at 37 °C with 5% CO2 in a cell culture incubator (Thermo Fisher, Waltham, MA, USA).

K562 cells are passaged in cell culture flasks (Corning, New York, NY, USA) every two days. Cells are harvested by centrifugation at 1400 rpm for 4 min using 50 mL centrifuge tubes. The supernatant is discarded, and cells are resuspended in fresh culture medium to a concentration of 10^4^ cells/mL before being returned to the incubator. In total, we prepare six samples with quantities ranging from 0.25 × 10^6^ cells/mL to 8 × 10^6^ cells/mL, in which the number of samples are increased by a factor of two. Cell counting is performed using a cell counter (Thermo Fisher, Waltham, MA, USA) with counting slides. The culture medium is transferred to 50 mL centrifuge tubes and centrifuged at 1400 rpm for 4 min. The cells are washed twice with PBS (Biological Industries, Beit Haemek, Israel). The cells are resuspended in PBS, counted, and accurately diluted to the desired concentration for subsequent experiments. As illustrated in Figure 3, cell images are captured using a microscope and camera system (Nikon, Tokyo, Japan). Cell counting is assessed using the Cell Counting Kit-8 (CCK-8, Beyotime, Shanghai, China). Ten microliters of cells from the counting procedure is plated in a 96-well plate (Corning, New York, NY, USA). To each well, 100 μL of culture medium and 10 μL of CCK-8 reagent are added, mixed thoroughly, and incubated in the cell culture incubator for 2 h. Absorbance at 450 nm is measured using a microplate reader (Bio-Rad, Hercules, CA, USA). Cell numbers are determined based on a standard curve. Cell images are captured using a microscope and camera system (Nikon, Tokyo, Japan). In addition, the S-parameter measurements are obtained using a vector network analyzer (VNA model) with a frequency range from 0.0001 GHz to 3.0001 GHz measured at a frequency step of 0.0003 GHz.

## 3. Results

### 3.1. Characterization and Experimental Process

**Figure 4:**
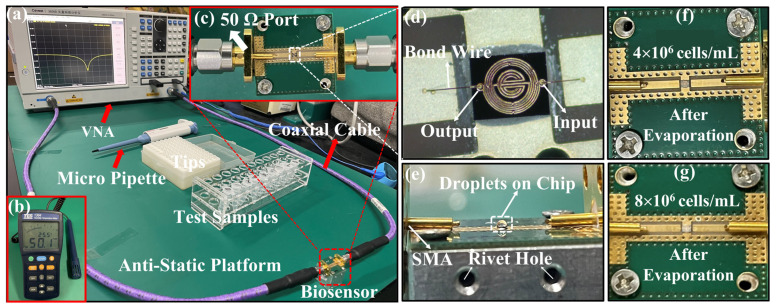
The experimental setup and preparation for the measurement of white blood cells and detailed information of proposed device. (**a**) Experimental setup; (**b**) the humidity–temperature meter; (**c**) the tested device connected to a VNA through a 50 Ω port; (**d**) enlarged view of the sensing chip with bonding wire; (**e**) side view of the sample as it drops on the chip; (**f**,**g**) represent the top views of chip with different number of residual cells.

The measurement platform in the biological laboratory is shown in Figure 4a, including a VNA (Ceyear, 3656B), the proposed microwave-sensing chip connected with the VNA via a coaxial cable, a pipette and its tip, WBC samples, an anti-static hand ring, and an anti-static platform. Figure 4c represents a top view of the device packed with a 50 Ω port impedance-matched connection to the VNA. Figure 4d shows a microfabrication diagram of the IPD chip. Before measuring the WBC solution, we record the S-parameter data of the die. Then, 1 μL PBS solution is pipetted onto the IPD chip, and the data are recorded. The side view after pipetting is shown in Figure 4e. Rivet holes are customized to allow the SMA (SubMiniature Version A) to contact the PCB (printed circuit board). These two sets of data above are used as the comparison group. The S-parameter response is recorded after the chip surface is cleaned and dried, and the WBC solution measurement is performed after it returns to the original value of the previously recorded bare chip. During the measurement of six sets of samples, 1 μL of a sample from the sample tube is pipetted for each measurement and added dropwise to the chip to give a spherical appearance. Then, the water molecules in the droplets on the chip begin to evaporate, and the volume of the spherical droplets gradually decreases, while the WBCs remain on the chip. After about ten minutes, the droplets naturally evaporate completely and the data are recorded. The chip is cleaned with PBS (phosphate buffered saline) solution and deionized water, then the surface is totally dried using a N_2_ gun, and the measurements are repeated twice after making it dry with absorbent paper and observing its scattering parameters back to the original values of the bare chip to minimize the error. Before the subsequent measurement, the pipette tip is replaced to prevent the cells left in the filter tip during the previous measurement from affecting the next measurement. The above steps are repeated for subsequent measurements. In addition, the temperature and relative humidity are kept at 26.00 ± 0.40 °C and 54.40 ± 3.90 RH% and recorded every half an hour throughout the measurement process. The short temperature and humidity range does not affect the measurement results [39]. Furthermore, due to the low output voltage of the VNA, the medium conductivity has little effect on the measurement results [40]. Finally, since evaporation measurements are made in this paper, the effect of ambient humidity on the measurement results is also almost negligible.

### 3.2. Sensing Response Analysis

**Figure 5:**
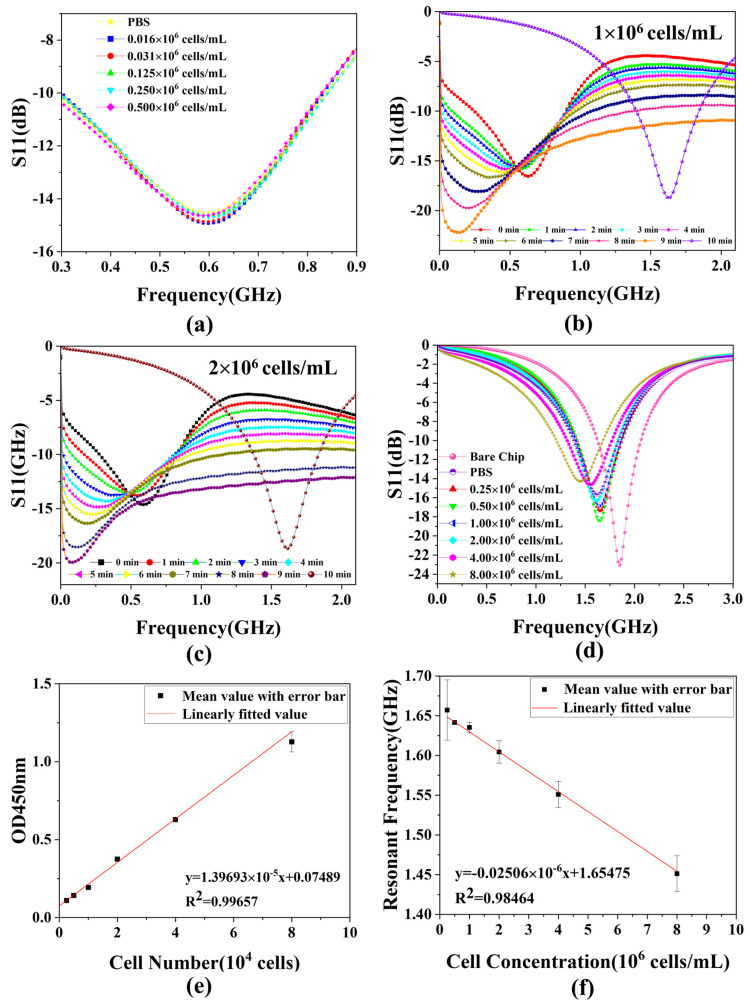
Data measured and analyzed at different WBC concentrations. (**a**) Measured reflection coefficient (S11) in pre-experiment; (**b**,**c**) S−parameter measurements of the whole evaporation process at two cell concentrations; (**d**) S11 characterization measured in formal experiments; (**e**) OD450 nm absorbance standard curve with error bar; (**f**) linear fit between cell number and resonant frequency with error bar. Note: error bars generated by fitting multiple measurement data using standard deviation (SD < 3.8%).

In this experiment, we choose S11 of the scattering parameters to characterize the experimental results because it is highly resistant to changes in the surrounding environment. The data from bare chips and PBS solutions that evaporated completely and naturally after titration are utilized to characterize the impact of introduced white blood cells on the biosensor. In the pre-experiment, as shown in Figure 5a, five sets of WBC solution samples of different concentrations and the PBS solution are measured using a pipette to aspirate 1 μL of the sample and then dropwise add it to the chip, making it spherical, followed by immediately recording the data of the S11 parameter shown on the VNA (<5 s). The results represent that the S11 curves of the five groups of WBC samples and PBS solutions overlap, indicating that they present the same characteristics through the sensor, verifying that the dielectric properties of WBC solutions and PBS are basically the same. Therefore, the method of recording the data immediately after the drop cannot reflect the difference between the different concentrations of cells. This is why we use the method of evaporation measurements. Figure 5b,c shows the S-parameter measurements of the whole evaporation process at two cell concentrations, 1 × 10^6^ cells/mL and 2 × 10^6^ cells/mL, respectively. As the evaporation time gradually increases, the resonant frequency gradually decreases, which is due to the fact that water evaporation corresponds to an increase in the concentration of the cell solution on the droplet, which increases its dielectric constant, leading to an increase in capacitance and thus a decrease in the resonant frequency. After the resonant frequency reaches its lowest value, the water molecules are completely evaporated, so the resonant frequency value increases rapidly to reach a stable value. This is the reason that the water completely evaporates and only cells remain on the surface of the chip at this time in the measurement of the cell’s dielectric properties, which is very different from the cells in the PBS solution, so the resonant frequency is also different. Since the curve on the VNA changes very rapidly during this period of time when the resonant frequency increases, it is not possible to record the complete process accurately manually, so only its final relatively stable value is recorded. As for the experiment measured by the evaporation method, the data of the reference and the data measured for each concentration are shown in Figure 5d. As the concentration of the WBC solution increases, the overall S11 curve gradually shifts to the left relative to the bare chip due to changes in the dielectric properties of the chip surface. This is the reason why, when the number of cells increases, the dielectric constant of the chip surface increases, the capacitance value increases, the resonant frequency decreases, and the S11 curve gradually shifts to the left. Moreover, the greater the difference in concentration, the larger the leftward shift. After the WBC solution is added to the chip surface and left to evaporate completely, the white blood cells remain on the chip, causing changes in the dielectric properties of the surface. The interaction between these cells and the electromagnetic waves generated by the microwave biosensor alters capacitance and inductance values, affecting resonant frequency. In addition, the number of cells remaining varies depending on the concentration of the WBC solution, so the impact on the dielectric properties of the chip surface is different, and the degree of offset of the S11 curve is not the same. As shown in Figure 4f,g, cells remain on the chip after the evaporation of the samples for the two highest cell concentrations. Therefore, the characteristics represented in the figure are consistent with the theory. In contrast, the data of the PBS solution after complete natural evaporation concern the position between the two cell concentrations of 10^6^ cells/mL and 2 × 10^6^ cells/mL. In order to maintain the activity and suspension of the measured WBC samples during the measurement process, they are typically suspended in PBS buffer to maintain isotonicity inside and outside the cells. The main components of the PBS solution are various phosphates. This implies that the dielectric constant characterized by a WBC is approximate to that of the PBS solution, verified in the pre-experiment, as shown in the illustration in Figure 5a.

Therefore, the PBS in the formal experiment after evaporation also shows similar S11 properties to a specific cell concentration, which explains why its S11 curve is located between the two cell concentrations. Figure 5f shows the relationship between cell concentrations and the value of resonant frequency. As the cell concentration increases, the resonant frequency gradually decreases. The cell concentration increases, the number of cells remaining on the chip increases, the dielectric constant of the chip surface increases, the capacitance value increases, and the resonant frequency decreases.

The average diameter of the K562 cells is about 15 μm, the maximum cross-sectional area is about 1.8 × 10^−10^ m^2^, and the surface area of the chip is about 1 mm × 1 mm, and so it can be approximated that about 5660 cells are needed to fill the surface of the chip. In fact, since the cells will be squeezing each other, the number will be more than the calculated value. As the number of cells increases, the vertical height increases with cell stacking, estimating the minimum height as the thickness of the two cell membranes and the maximum height as the sum of the diameters of the two cells. The final height range estimated is 0.02–30 μm, which is within the radiation range of the sensor and can be detected.

Ideally, based on the gradient, the number of cells deposited on the chip can be considered as 250, 500, 1000, 2000, 4000, and 8000, and they exhibit more pronounced differences in S11 characteristics. This also indicates that the proposed biosensor possesses high sensitivity in both the planar and vertical directions, capable of responding to changes in cell size.

### 3.3. Linearization of Derived Parameters

For microwave sensors, the linearization of the measured S-parameters gives a better result after the interacting of the cell sample with the electromagnetic wave. In order to obtain accurate cell counts, optical methods are first used to measure the cell’s 450 nm absorbance, which can be used as a more accurate reference standard for cell counts. After obtaining the resonant frequency corresponding to each group of WBC concentration, the relationship between different WBC concentrations and resonant frequency is analyzed by linear regression, as shown in Figure 5f. As can be seen from the figure, there is a good correlation with the linear fit of *r*^2^ = 0.98464 (*r* = correlation coefficient) between the resonant frequency and WBC concentration, and the fitted curve can be expressed as follows:

(6) $$y=-0.02506\times 10^{-6}x-1.65475,$$

where y represents the resonant frequency shift and x represents the concentration of the WBC solution. The above relationship indicates that the proposed microwave biosensor has a high sensitivity of 25.06 Hz/cells·mL^−1^ for a quantitative sample of 1 μL. In addition, comparing Figure 5e,f, the curves measured by the microwave biosensor are essentially the same as those measured by the well-established optical method, which also demonstrates the accuracy of the microwave biosensor in measuring the number of white blood cells.

### 3.4. Microscopic Sensing Mechanisms

Biomaterials have unique dielectric properties. The interaction of electromagnetic fields with biological tissues is related to their dielectric properties. The electromagnetic field exerts two types of controlling dielectric behaviors on biological tissues, namely, the oscillation of free charges or ions and the rotation of dipole molecules under the applied electromagnetic energy frequency. The first type results in conduction currents with associated energy losses due to the electrical resistance of the medium, while the other type influences displacement currents through the medium and associated dielectric losses due to viscosity. These effects can affect the magnitude of the dielectric constant [41]. The dielectric properties of the cell consist mainly of the cell membrane and the cytoplasm. The cell membrane is a thin film on the cell’s surface that controls the exchange and transportation of substances in and out of the cell. The cytoplasm contains various salt ions, polar protein molecules, and polar water molecules. Polar molecules do not show polarity without an applied electric field. When polar molecules are placed in an applied electric field, each polar molecule will form an orderly arrangement along the direction of the electric field force and induce an opposite charge on the surface of the dielectric. The stronger the applied electric field, the stronger the polarization. When the applied electric field changes direction, the polar molecules also form an orderly arrangement in the opposite direction. In the case of polar molecules (which already have a net permanent dipole), such as water, the randomly distributed molecular dipoles rotate in the direction of the electric field, producing a net polarization in that direction. This is known as depolarization or directional polarization. In addition, at low-frequency fields, there may be charge carriers that are able to migrate some distance by diffusion or jumping through most materials. In biological tissues, this occurs at the cell membrane. This type of polarization is known as space charge, interfacial, or Maxwell–Wagner polarization [42].

**Figure 6:**
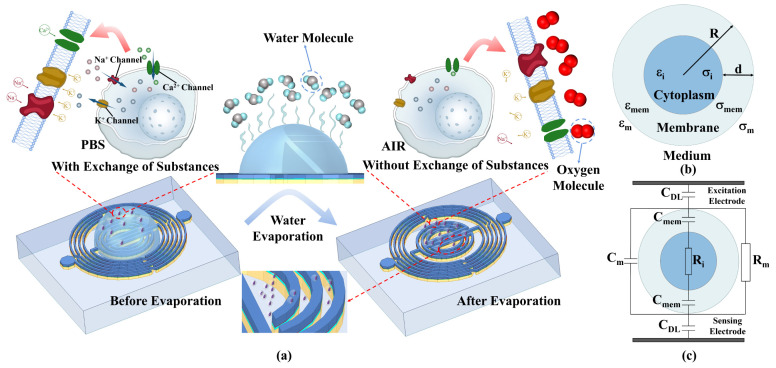
(**a**) Changes occurring in the cells before and after complete evaporation of the solution; (**b**) modeling of individual cell and (**c**) its equivalent circuit.

Figure 6a represents the different states of the cells before and after evaporation. A single cell in suspension is modeled as shown in Figure 6b. Here, $\varepsilon_{\mathrm{mem}}$ and $\varepsilon_{i}$ are the complex dielectric constants of the cell membrane and cytoplasm, respectively. *R* and *d* (*d* < < *R*) are the cell’s radius and membrane thickness, respectively. For a single-shell spherical cell model, $\varepsilon_{P}$ is [43]:

(7) $$\varepsilon_{P}=\varepsilon_{\mathrm{mem}}\frac{\gamma^{3}+2\left(\frac{\varepsilon_{i}-\varepsilon_{\mathrm{mem}}}{\varepsilon_{i}+2\varepsilon_{\mathrm{mem}}}\right)}{\gamma^{3}-\left(\frac{\varepsilon_{i}-\varepsilon_{\mathrm{mem}}}{\varepsilon_{i}+2\varepsilon_{\mathrm{mem}}}\right)},$$

with $\gamma =R/\left(R-d\right)$. Figure 6c shows the equivalent circuit for this modeling. A resistor and a capacitor in parallel represent the impedance of the medium. The cell membrane of a living cell has very low conductivity and can be modeled as a capacitor, and a resistance with negligible capacitance represents the cytoplasm. In addition, the electrode–electrolyte interface impedance is modeled as a capacitor, CDL, referred to as the electric double layer (EDL) effect. Then, the system’s response can be clearly illustrated in different frequency ranges. Due to the presence of the EDL, the detection sensitivity is low at very low frequencies (<1 kHz), and most of the voltage drops in the EDL, which does not provide information about cell properties (inside and outside the cell membrane). At low frequencies (<1 MHz), the cell membrane undergoes polarization, $C_{\mathrm{mem}}$ acts as an insulating layer, and the current flows only in the extracellular medium. Furthermore, since the current does not pass through the cell, it remains impossible to obtain information about the inside of the cell. When the frequency is increased (1–100 MHz), the capacitive reactance $C_{\mathrm{mem}}$ of the cell decreases, increasing the intracellular current and thus the conductivity. A further increase in frequency (>100 MHz) short-circuits $C_{\mathrm{mem}}$ effectively, and the capacitive barrier of the cell membrane may be crossed, allowing the electric field to pass through the cytoplasm. At these frequencies, the signal becomes sensitive to changes in intracellular material and reaches maximum conductivity [43,44]. The proposed biosensor operates in a frequency band greater than 100 MHz, so it can pass through the cell membrane to detect intra-membrane substances instead of the cells. Cells in PBS solution need to maintain viability, meaning substances need to exchange between the inside and outside of the cell membrane to maintain isotonicity. As a result, the substances inside the membrane and the external PBS solution exhibit similar dielectric properties. It is theoretically explainable that the separate cell membrane relative to the membrane inside and outside of the material shows that the dielectric properties are minimal, which leads to different cell concentrations dropping on the chip when the S11 characteristics are the same. When the solvent is completely and naturally evaporated and the cells are exposed to air, there is no exchange of material between the inside and outside of the membrane, and the material inside the membrane exhibits dielectric properties that are very different from the air. At this time, microwave can detect the presence of cells in the air. The number of cells remaining in the cell solution at different concentrations is also different; the final S11 characteristics are also different. Based on the change in cell concentration, the resonant frequency also shows a corresponding change. A comparative analysis with the previous experiments that have already been conducted to detect the biological samples is shown in Table 1. Among them, the detection method and some features of this study are advanced and innovative. In this work, a microwave sensor fabricated by IPD technology and using evaporation for WBC counting is proposed, being of small size and possessing high sensitivity and good linearity. There are also other methods that detect the WBC counts. Paper-based vertical flow platforms capable of WBC counting require the use of gold nanoparticles to label the cells first, which has a certain technological threshold and requires a certain amount of time for staining. In addition, microorganisms and cells have similar structural compositions, and microwave methods are also commonly used to detect *E. coli*. Microwave sensors with multi-resonant circuit and planar single and dual-resonant microwave biosensors have been used to detect *E. coli* counts, with good sensitivity but poor linearity, which is not conducive to the development of subsequent matching circuits. The use of blood smear image automatic recognition for WBC counting has certain requirements for computer configuration, which is not conducive to use in some specific occasions. Therefore, the microwave detection of a WBC count proposed in this paper is characterized by good linearity and a wide range of occasions for use.

## 4. Conclusions

A highly sensitive and linear permittivity-inspired microwave sensor is proposed to detect the number of white blood cells. We employ an innovative experimental measurement method to record the data after the cell solution dripped on the chip is completely evaporated naturally. The biosensor assayed different cell concentrations with good linearity. The experimental results verify that the dielectric properties of the WBC solution are essentially the same as those of the PBS solution in microwave detection. The microscopic sensing mechanisms are also further analyzed and explained regarding the difference between the environment inside and outside the cell membrane. An extension of the current work is to achieve faster solution evaporation without affecting the experimental results, building subsequent matching circuits, and reducing the equipment size used to create a small integrated platform for a convenient WBC count. Overall, this microwave method provides a potential tool for portable WBC counting, such as in emergency situations where large devices cannot be used or suspected cases need to be diagnosed in a timely manner, and it validates the widespread application of microwave sensing. For practical applications, the proposed biosensor could potentially be regarded as a calibration or measurement tool in hospitals or research facilities. Furthermore, it can be integrated into a portable cell-counting platform to be used for medical observations at home.

## Figures and Tables

**Table 1:** Performance comparison to other previously reported methods for detecting biological samples.

References	Sample	Structure	Sensing Method	Size (mm × mm)	Concentration	Sensitivity
[3]	WBC	Vertical flow configuration	Gold nanoparticl-es	NA	6 × 10^4^–16 × 10^4^cells/mL	NA
[45]	Escherichia coli	Multi-resonator	*f*r	12.00 × 15.00	10^3^–10^9^ CFU/mL	4.2 Hz/CFU/mL
[46]	Escherichia coli	LC-resonator	*f*r	2.01 × 6.60	10^3^–10^9^ CFU/mL	120 M Hz/CFU/mL
[47]	WBC	Blood smear images	svm	NA	NA	99.73% (average accuracy)
[48]	Blood cell	Optofluidic cell-counting platform	Image processing	NA	NA	NA
[49]	On-line cell/particle	Micro flow cytometers with integrated fiber optics	Electrokine- tically driven	NA	NA	NA
**Proposed biosensor**	**WBC**	**LC resonator**	***f*r, S11**	**1.00 × 1.00**	**0.25 × 10^6^–8 × 10^6^** **cells/mL**	**25.06 Hz/cells·mL^−1^**

CFU: colony-forming units; NA: not available.

## Data Availability

Data are contained within the article.
